# Clinical assessment of resting full-cycle ratio and fractional flow reserve for coronary artery disease in a real-world cohort

**DOI:** 10.3389/fcvm.2022.988820

**Published:** 2022-10-25

**Authors:** Ming-Ju Chuang, Chun-Chin Chang, Yin-Hao Lee, Ya-Wen Lu, Yi-Lin Tsai, Ruey-Hsing Chou, Cheng-Hsueh Wu, Tse-Min Lu, Po-Hsun Huang

**Affiliations:** ^1^Division of Cardiology, Department of Medicine, Taipei Veterans General Hospital, Taipei, Taiwan; ^2^Cardiovascular Research Center, National Yang-Ming Chiao Tung University, Taipei, Taiwan; ^3^Institute of Clinical Medicine, National Yang-Ming Chiao Tung University, Taipei, Taiwan; ^4^Department of Cardiology, Thoraxcenter, Erasmus Medical Center, Rotterdam, Netherlands; ^5^Department of Critical Care Medicine, Taipei Veterans General Hospital, Taipei, Taiwan; ^6^Healthcare and Services Center, Taipei Veterans General Hospital, Taipei, Taiwan

**Keywords:** fractional flow reserve, resting full-cycle ratio, coronary artery disease, percutaneous coronary intervention, coronary physiology

## Abstract

**Background:**

There are few reports published on the comparison of the resting full-cycle ratio (RFR) and fractional flow reserve (FFR) on the assessment of the severity of coronary stenosis. We aimed to investigate the diagnostic accuracy of RFR for detection of functionally significant coronary lesions.

**Methods:**

This was an observational, retrospective, single-center study. We evaluated both RFR and FFR for 277 coronary lesions of 235 patients who underwent coronary angiography. Patients presenting with chronic coronary syndrome, unstable angina, or non-ST-elevation myocardial infarction were included.

**Results:**

The mean FFR and RFR values were 0.84 ± 0.08 and 0.90 ± 0.08, respectively. RFR significantly correlated with FFR (*r* = 0.727, *P* < 0.001). The agreement rate between the FFR and RFR was 79.8% (221/277). The diagnostic performance of RFR vs. FFR was accuracy 79.8%, sensitivity 70.4%, specificity 83.7%, positive predictive value 64.0%, and negative predictive value 87.2%. The discriminative power of RFR to identify lesions with FFR ≤ 0.80 was acceptable when the RFR value was within the gray zone [0.86 ≤ RFR ≤ 0.93; AUC: 0.72 (95% CI:0.63–0.81)], while it was excellent when the RFR value was out of the gray zone [RFR > 0.93 or < 0.86; AUC: 0.94 (95% CI:0.88–0.99)].

**Conclusion:**

RFR was significantly correlated with FFR in the assessment of intermediate coronary stenosis. An RFR-FFR hybrid approach increases the diagnostic accuracy of RFR in the detection of functionally significant lesions.

## Introduction

Fractional flow reserve (FFR) is a pressure wire-based physiology index used to assess coronary stenoses under maximal hyperemia. Currently, the use of FFR to guide coronary revascularization has been assigned a class I (level of evidence A) indication in the European guidelines (1). The Fractional Flow Reserve vs. Angiography for Multivessel Evaluation (2) (FAME) and FAME 2 studies (3) have demonstrated that the FFR-guided percutaneous coronary intervention (PCI) strategy is associated with a significantly lower rate of cardiovascular events in patients with stable coronary artery disease. Despite accumulating evidence to support the use of FFR, the adoption rate of FFR remains limited in clinical practice for several reasons, such as adenosine-related side effects, prolonged procedure time, additional equipment needed, and cost-related issues (4).

Several non-hyperemic physiological indices have been developed to avoid the side effects of adenosine and reduce the procedural aspects of physiology assessment, including the instantaneous wave-free ratio (iFR) (5–8) and resting full-cycle ratio (RFR)(9). It has been confirmed in randomized control trials that iFR-guided revascularization was non-inferior to FFR-guided revascularization in terms of the risk of major adverse cardiac events (10, 11). RFR was developed to detect the lowest distal coronary pressure (Pd) to aortic pressure (Pa) ratio (Pd/Pa) within the entire cardiac cycle. The VALIDATE RFR study (9) reported that the diagnostic accuracy of RFR was equivalent to that of iFR and the lowest Pd/Pa ratio was outside of diastole in 12% of all cardiac cycles, suggesting that RFR may detect clinically significant lesions that would be missed by other methods dedicated to a specific segment of the cardiac cycle.

Nevertheless, the correlation between RFR and FFR on functional assessment has rarely been reported. Therefore, this study aimed to investigate the correlation between pressure wire-based FFR and RFR in the functional assessment of coronary stenoses in the real-world practice.

## Materials and methods

### Study design and participants

The present study was an observational, retrospective, single-center study that compared FFR and RFR. Between May 01, 2019, and May 24, 2022, patients presenting with chronic coronary syndrome, unstable angina, or non-ST elevation myocardial infarction who underwent both RFR and FFR assessment at baseline were eligible. The physiological assessment was performed at the operator’s discretion based on the coronary angiograms and clinical information. Coronary physiology was assessed for intermediate-degree of stenosis defined by visual assessment. In patients with acute coronary syndrome, RFR and FFR were performed for the assessment of non-culprit lesions.

### Coronary physiology assessment

RFR and FFR were performed according to the standard local clinical practice in the catheterization laboratory. RFR was measured using a 0.014 PressureWire™ X Guidewire (Abbott Vascular Inc., Santa Clara, CA, USA) positioned distal to the target lesion. The QUANTIEN™ or OPTIS™ Integrated System (Abbott Vascular Inc., Santa Clara, CA, USA) was used for measurement. RFR was defined as the lowest Pd/Pa ratio during the entire cardiac cycle (9). After RFR measurement, FFR was measured under hyperemia by intravenous (IV) adenosine infusion or intracoronary (IC) bolus injection of adenosine. For IV adenosine infusion, an injection pump was used to achieve a dose of 140 μg/kg/min continuously for 3 min through a large cubital or femoral vein. For IC adenosine injection, the dose of IC adenosine was initiated from 60 to 120 μg for the right coronary artery (RCA) and 120 μg for the left coronary artery. The dose of IC adenosine was escalated subsequently (180, 240, 360, or 480 μg) and at least two increasing doses of IC adenosine were used to achieve hyperemia when FFR > 0.80. The values of FFR/RFR were adjudicated by two interventionalists.

### Clinical endpoints

The primary endpoint of this study was the vessel-oriented composite endpoint (VOCE) during clinical follow-up, defined as a composite of all-cause death, vessel related non-fatal myocardial infarction (MI), and target vessel revascularization (TVR). MI was defined according to the Third Universal Myocardial Infarction definition (12). TVR was defined as any repeat percutaneous intervention or surgical bypass of the target vessel. VOCE was analyzed hierarchically.

### Statistical methods

Categorical variables are presented as percentages and numbers. Continuous variables are presented as the mean ± standard deviation. Spearman’s correlation coefficient was used to evaluate the relationship between the FFR and RFR. The receiver operating characteristic curve and the area under the curve (AUC) were used to estimate the discriminative value of RFR for functionally significant coronary lesions (FFR ≤ 0.80). Survival curves were constructed using the Kaplan-Meier estimates. A two-sided *p*-value of less than 0.05 was considered to indicate statistical significance. Data were analyzed using SPSS software (version 25, SPSS, Chicago, IL, USA).

## Results

A total of 277 lesions from 235 patients were included in the analysis. The baseline characteristics of eligible patients are shown in Table 1. The mean age was 67.8 ± 10.5 years, 77.9% were male, 40.4% had diabetes, 54.5% presented with chronic coronary syndrome, and 40% had unstable angina. Coronary stenoses were mainly located in the left anterior descending artery (70.7%). The majority (56.6%) of lesions had diameter stenosis between 50 and 70% by visual assessment. The mean FFR and RFR values were 0.84 ± 0.08 and 0.90 ± 0.08, respectively (Table 2).

**TABLE 1:** Baseline characteristics of patients.

Baseline characteristics	All patients (*n* = 235)
Age (years)	67.8 ± 10.5
**Sex**	
Male	183 (77.9%)
Female	52 (22.1%)
**Medical history**	
Hypertension	178 (75.7%)
Diabetes mellitus	95 (40.4%)
Peripheral vascular disease	6 (2.6%)
Chronic kidney disease	48 (20.4%)
Previous myocardial infarction	18 (7.7%)
Previous percutaneous coronary intervention	65 (27.7%)
Previous coronary artery bypass grafting	5 (2.1%)
**Clinical presentation**	
Chronic coronary syndrome	128 (54.5%)
Unstable angina	99 (42.1%)
Non-ST elevation myocardial infarction	8 (3.4%)
**Number of diseased vessels**	
Single-vessel disease	112 (47.7%)
Two-vessel disease	75 (31.9%)
Three-vessel disease	48 (20.4%)

**TABLE 2:** Baseline characteristics of vessels studied.

	All vessels (*n* = 277)
**Vessel studied**	
Left main	1 (0.4%)
Left anterior descending artery	196 (70.7%)
Left circumflex	37 (13.4%)
Right coronary artery	43 (15.5%)
**Lesion severity**	
Mean diameter stenosis (visual assessment)	63 ± 13%
Stenosis ≥ 70%	106 (38.3%)
50% ≤ stenosis < 70%	157 (56.7%)
Stenosis < 50%	14 (5.0%)
**FFR value**	
Mean ± SD	0.84 ± 0.08
Median (IQR)	0.85 (0.79, 0.89)
>0.80	196 (70.8%)
≤0.80	81 (29.2%)
**RFR value**	
Mean ± SD	0.90 ± 0.08
Median (IQR)	0.91 (0.88, 0.95)
>0.89	188 (67.9%)
≤0.89	89 (32.1%)
PCI was performed actually	93 (33.6%)

FFR, fractional flow reserve; RFR, resting full-cycle ratio.

**FIGURE 1:**
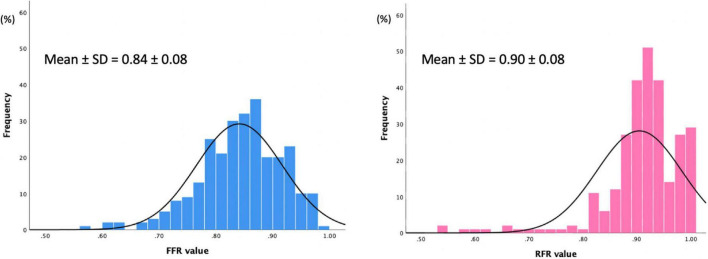
Distribution of values for FFR and RFR.

**FIGURE 2:**
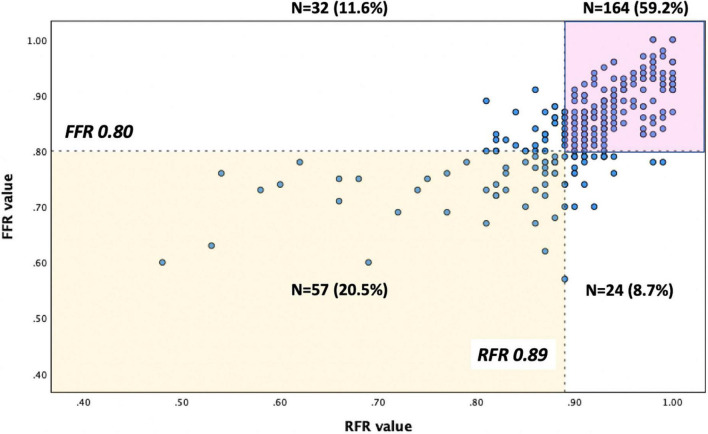
Correlation between FFR and RFR.

The distributions of FFR and RFR values are shown in Figure 1. RFR was highly positively correlated with FFR (*r* = 0.727, *P* < 0.001) (Figure 2). Using the cut-off value of RFR ≤ 0.89 to define a functionally significant lesion, the diagnostic concordance between RFR and FFR was 79.8% (Table 3). The univariate and multivariate regression analysis were performed to identify predictors of discordance between RFR and FFR. Prior MI [Odds Ratio (OR), 3.39; 95% confidence interval (CI), 0.89–12.9; *p* = 0.074] and LAD lesion (OR 2.10; 95% CI, 0.95–4.55, *p* = 0.067) tended to be predictors of RFR/FFR discordance (Table 4).

**TABLE 3:** Agreement between FFR and RFR.

	FFR ≤ 0.8	FFR > 0.8	Total
RFR ≤ 0.89	57 (20.5%)	32 (11.6%)	89 (32.1%)
RFR > 0.89	24 (8.7%)	164 (59.2%)	188 (67.9%)
Total	81 (29.2%)	196 (70.8%)	277

Data was number (%). FFR, fractional flow reserve; RFR, resting full-cycle ratio.

**TABLE 4:** Predictors of RFR and FFR discordance.

Variables	Univariate analysis Odds ratio 95%CI	*P*-value	Multivariate analysis* Odds ratio 95%CI	*P*-value
Age	1.01 (0.98–1.03)	0.704		
Woman	1.38 (0.69–2.75)	0.361	1.29 (0.63–2.67)	0.490
Hypertension	0.77 (0.40–1.49)	0.434	0.72 (0.36–1.45)	0.359
Diabetes mellitus	1.24 (0.69–2.23)	0.478	1.44 (0.76–2.75)	0.264
Chronic kidney disease	1.35 (0.69–2.66)	0.377	1.28 (0.99–5.23)	0.500
Peripheral vascular disease	1.13 (0.23–5.60)	0.879		
Previous MI	1.57 (0.54–4.60)	0.412	3.39 (0.89–12.9)	0.074
Previous PCI	0.60 (0.30–1.21)	0.153	0.48 (0.19–1.20)	0.115
LAD lesions	2.18 (0.76–4.84)	0.039	2.10 (0.95–4.55)	0.067
Acute coronary syndrome	0.91 (0.50–1.64)	0.748		

*Variables with *p* < 0.50 in the univariate analysis were included in the multivariate regression analysis.

The diagnostic performance of RFR vs. FFR was reported to have a diagnostic accuracy of 79.8%, sensitivity 70.4%, specificity 83.7%, positive predictive value 64.0%, and negative predictive value 87.2%. A sub-analysis was performed to identify predictors of false positive RFR results (RFR ≤ 0.89/FFR > 0.80). Chronic kidney disease tended to be a predictor of low RFR/high FFR (OR 2.34; 95% CI, 1.27–5.92, *p* = 0.052) (Table 5).

**TABLE 5:** Predictors of false positive RFR result (RFR ≤ 0.89/FFR > 0.80).

Variables	Univariate analysis Odds ratio 95%CI	*P*-value	Multivariate analysis* Odds ratio 95%CI	*P*-value
Age	1.03 (0.99–1.07)	0.075	1.02 (0.98–1.06)	0.276
Woman	2.27 (1.02–5.03)	0.044	2.23 (0.94–5.28)	0.069
Hypertension	0.81 (0.36–1.85)	0.618		
Diabetes mellitus	1.90 (0.90–3.99)	0.092	1.64 (0.72–3.78)	0.240
Chronic kidney disease	2.74 (1.27–5.92)	0.011	2.34 (1.27–5.92)	0.052
Peripheral vascular disease	2.27 (0.45–11.4)	0.321	2.18 (0.38–12.5)	0.382
Previous MI	0.43 (0.06–3.37)	0.423	0.58 (0.06–5.59)	0.640
Previous PCI	0.65 (0.27–1.57)	0.333	0.64 (0.22–1.83)	0.404
LAD lesions	1.91 (1.04–4.57)	0.171	1.78 (0.65–4.90)	0.265
Acute coronary syndrome	0.71 (0.33–1.52)	0.381	0.64 (0.28–1.47)	0.290

*Variables with *p* < 0.50 in the univariate analysis were included in the multivariate regression analysis.

**FIGURE 3:**
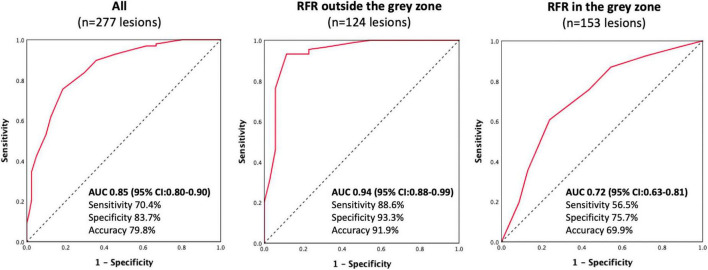
Receiver operating characteristic curves.

The discriminative power of RFR for functionally significant coronary lesions (FFR ≤ 0.80) was excellent. AUC was 0.85 (95% CI:0.80–0.90) (Figure 3). A total of 153 lesions had an RFR value in the gray zone (0.86 ≤ RFR ≤ 0.93), while 124 lesions had an RFR value outside the gray zone (RFR < 0.86 or RFR > 0.93). The diagnostic performance of RFR was excellent for FFR ≤ 0.80, when RFR was outside the gray zone [AUC: 0.94 (95% CI: 0.88–0.99), *P* < 0.0001]. However, the diagnostic performance of RFR was only acceptable when RFR was in the gray zone [AUC: 0.72 (95% CI: 0.63–0.81), *P* < 0.0001], assuming that FFR is always accurate.

**FIGURE 4:**
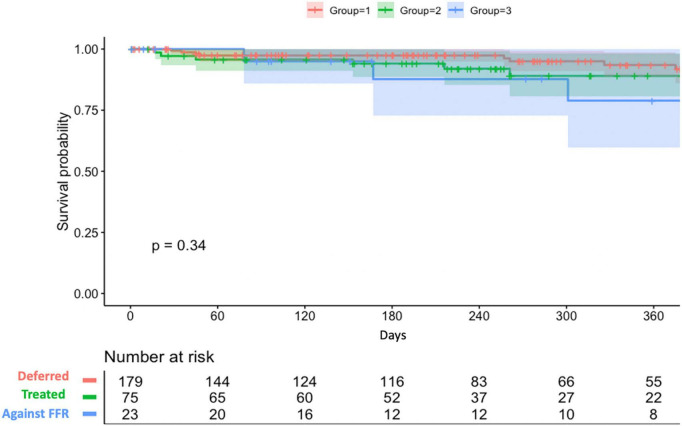
VOCE-free survival curves.

Overall, 91.7% (254/277) of lesions were treated according to their RFR and FFR result (179 lesions were deferred and 75 lesions were treated by PCI). On the other hand, 23 lesions were treated against to FFR result (PCI was done for 19 lesions with FFR > 0.80 and 4 lesions with FFR ≤ 0.80 were only under medical therapy). Among 94 lesions treated by PCI, post-PCI functional assessment was performed for 25 lesions (26.6%). The mean post-PCI FFR and RFR values were 0.87 and 0.91, respectively. Clinical outcomes stratified by actual treatments were shown in Table 6. During a mean follow-up of 258 ± 182 days, the VOCE-free survival rate was numerically higher in the deferred group than the treated group and against FFR group (93.5% vs. 89% vs. 78.9%, *P* = 0.34) (Figure 4).

**TABLE 6:** Clinical outcomes stratified by treatment strategy.

	Deferred *N* = 179	Performed *N* = 75	Against FFR *N* = 23	*P*-value
VOCE	10 (5.6%)	7 (9.3%)	3 (13.0%)	0.305
Death	5 (2.8%)	3 (4.0%)	0 (0.0%)	0.600
Non-fatal MI	1 (0.6%)	0 (0.0%)	0 (0.0%)	0.760
TVR	6 (3.4%)	4 (5.3%)	3 (13.0%)	0.112

VOCE, vessel-oriented composite endpoint; MI, myocardial infarction; TVR, target vessel revascularization.

## Discussion

The present study investigated the diagnostic accuracy of RFR in detecting functionally significant lesions (FFR ≤ 0.80) in patients with chronic coronary syndrome, unstable angina, or non-ST elevation myocardial infarction. The main findings were as follows: (1) RFR had a positive correlation with the FFR. (2) The concordance rate between RFR and FFR was high (79.8%) in our study. (3) The discriminative power of RFR to identify lesions with FFR ≤ 0.80, was acceptable when the RFR value was within the gray zone (0.86 ≤ RFR ≤ 0.93), while it was excellent when the RFR value was outside the gray zone (RFR > 0.93, or < 0.86).

Accumulating data supports the use of FFR to guide revascularization of chronic or acute coronary syndrome (13, 14). To date, non-hyperemic physiological indices, such as iFR and RFR, have been invented to avoid the side effects of adenosine and reduce the procedural time of physiological assessment (9, 10). Previous studies showed that a hybrid iFR-FFR approach for revascularization could increase the adoption of physiology-guided PCI and still have a high agreement with the FFR-only strategy (15, 16). Similarly, our study demonstrated that when the RFR value was in the gray zone, evaluation of FFR was suggested to confirm the result to guide decision-making. When the RFR value was outside the gray zone, the RFR-only strategy could be used for deferring or performing PCI. An RFR-FFR hybrid approach can increase the diagnostic accuracy of RFR in detecting functionally significant lesions and may facilitate the physiological assessment of coronary lesions by simplifying the procedures. Otherwise, a RFR-only strategy with a treatment cut-off of ≤ 0.89 would have resulted in 11.6% of unnecessary PCI and 8.7% of lesions inappropriately deferred in our study cohort. In our study, FFR was almost mandatory following the initial RFR assessment, irrespective of the result. This early experience of our single-center may reflect the fact that RFR is a newly developed physiological index with relatively limited evidence. In fact, adenosine injection can be waived for 44.7% of lesions in our cohort, which can be simply deferred or treated by PCI based on the RFR results only.

Recently, the Resting-Full-Cycle Ratio Comparison vs. Fractional Flow Reserve study provided prospective validation data (17). In the abovementioned study, the diagnostic performance of RFR was diagnostic accuracy 79.0%, sensitivity 76.0%, specificity 80.0%, positive predictive value 68.0%, and negative predictive value 86.0%. These numbers are consistent with our findings and another report (18). It is noteworthy that the positive predictive value of RFR was slightly lower than expected and resulted in false positive result (RFR ≤ 0.89/FFR > 0.80). Wienemann et al. reported that LAD lesions, peripheral artery disease, age, woman and non-focal stenoses were predictors of false positive result of RFR (18). It has been known that several characteristics, such as chronic kidney disease or hemodialysis, affecting coronary flow reserve through microvascular function can influence resting and hyperremic coronary flow velocity (19, 20).

Compared to iFR and FFR, the concordance between RFR and FFR has rarely been reported in the literature. Lee et al. reported that the discrepancy rate between iFR and FFR was approximately 12% of the vessel analyzed, and it was mostly observed in the left anterior descending artery (LAD) (21). Likewise, Goto et al. showed that LM and LAD location, hemodialysis, and peripheral artery disease were associated with a low RFR among patients with a high FFR value (22). Similar findings were observed in our study. The discrepancies between RFR and FFR were 23.4, 8.1, and 13.9% for the LAD, left circumflex artery, and RCA, respectively. The potential explanation is that the large myocardial territory supplied by LM/LAD vessel compared to non-LAD vessels may cause higher coronary flow variation between resting and hyperemic status, which could be responsible for the higher discrepancy rate between resting and hyperemic indices in LM/LAD lesions (23). In our study, the presence of prior MI tended to be a predictor of RFR and FFR discordance. It has been shown that FFR value depends on the mass of viable myocardium not only the degree of coronary stenosis (24). Whether myocardial scarring after infarction may result in discrepancy between resting and hyperemic physiology indices need to be further explored.

Nevertheless, it has been reported that the discrepancy between resting physiological indices and FFR was not associated with the risk of vessel-oriented composite outcomes in deferred lesions. Only when lesions with concordant abnormal results in both resting physiological indices and FFR showed an increased risk of vessel-oriented composite outcomes (25).

### Limitations

The present study has some important limitations. First, this was a retrospective observational study, and data were obtained in a real-world practice without an independent core lab for pressure waveform interrogation. Second, the use of FFR or RFR was at the discretion of the treating physicians, and potential selection bias could not be avoided in this setting. Third, the reproducibility of RFR measurement has not yet been reported and warrants further investigation. Lastly, the results from a single-center experience with a limited sample size need to be interpreted cautiously. The adverse event rate was too low to detect any substantial differences.

## Conclusion

RFR is significantly correlated with FFR in the assessment of intermediate coronary stenoses. A hybrid approach increases the diagnostic accuracy of RFR for detecting functionally significant lesions.

## Data availability statement

The original contributions presented in the study are included in the article/supplementary material, further inquiries can be directed to corresponding author, C-CC.

## Ethics statement

The studies involving human participants were reviewed and approved by the Research Ethics Committee of the Taipei Veterans General Hospital. Written informed consent for participation was not required for this study in accordance with the national legislation and the institutional requirements. Written informed consent for participation was not required for this study in accordance with the national legislation and the institutional requirements.

## Author contributions

C-CC and P-HH contributed to the conception and design of the study. C-CC, Y-HL, M-JC, Y-WL, Y-LT, R-HC, C-HW, and T-ML contributed to data collection. C-CC and M-JC analyzed and interpreted the data and drafted the report, which was critically revised for important intellectual content by T-ML and P-HH. All authors participated in the work and reviewed and agreed with the content of the article, and approved the final version of the report.

## References

[1] Neumann FJ Sousa-Uva M Ahlsson A Alfonso F Banning AP Benedetto U 2018 ESC/EACTS guidelines on myocardial revascularization. *EuroIntervention.* (2019) 14:1435–534. 30667361 10.4244/EIJY19M01_01

[2] Tonino PA De Bruyne B Pijls NH Siebert U Ikeno F van’ t Veer M Fractional flow reserve vs. angiography for guiding percutaneous coronary intervention. *N Engl J Med.* (2009) 360:213–24. 19144937 10.1056/NEJMoa0807611

[3] De Bruyne B Fearon WF Pijls NH Barbato E Tonino P Piroth Z Fractional flow reserve-guided PCI for stable coronary artery disease. *N Engl J Med.* (2014) 371:1208–17. 25176289 10.1056/NEJMoa1408758

[4] Gotberg M Cook CM Sen S Nijjer S Escaned J Davies JE. The evolving future of instantaneous wave-free ratio and fractional flow reserve. *J Am Coll Cardiol.* (2017) 70:1379–402. 28882237 10.1016/j.jacc.2017.07.770

[5] Sen S Escaned J Malik IS Mikhail GW Foale RA Mila R Development and validation of a new adenosine-independent index of stenosis severity from coronary wave-intensity analysis: results of the ADVISE (ADenosine vasodilator independent stenosis evaluation) study. *J Am Coll Cardiol.* (2012) 59:1392–402. 10.1016/j.jacc.2011.11.003 22154731

[6] Berry C van ‘t Veer M Witt N Kala P Bocek O Pyxaras SA VERIFY (VERification of instantaneous wave-free ratio and fractional flow reserve for the assessment of coronary artery stenosis severity in everyday practice): a multicenter study in consecutive patients. *J Am Coll Cardiol.* (2013) 61:1421–7. 10.1016/j.jacc.2012.09.065 23395076

[7] Jeremias A Maehara A Genereux P Asrress KN Berry C De Bruyne B Multicenter core laboratory comparison of the instantaneous wave-free ratio and resting Pd/Pa with fractional flow reserve: the RESOLVE study. *J Am Coll Cardiol.* (2014) 63:1253–61. 10.1016/j.jacc.2013.09.060 24211503

[8] Escaned J Echavarria-Pinto M Garcia-Garcia HM van de Hoef TP de Vries T Kaul P Prospective assessment of the diagnostic accuracy of instantaneous wave-free ratio to assess coronary stenosis relevance: results of ADVISE II international, multicenter study (ADenosine vasodilator independent stenosis evaluation II). *JACC Cardiovasc Interv.* (2015) 8:824–33. 10.1016/j.jcin.2015.01.029 25999106

[9] Svanerud J Ahn JM Jeremias A van ‘t Veer M Gore A Maehara A Validation of a novel non-hyperaemic index of coronary artery stenosis severity: the resting full-cycle ratio (VALIDATE RFR) study. *EuroIntervention.* (2018) 14:806–14. 10.4244/EIJ-D-18-00342 29790478

[10] Davies JE Sen S Dehbi HM Al-Lamee R Petraco R Nijjer SS Use of the Instantaneous wave-free ratio or fractional flow reserve in PCI. *N Engl J Med.* (2017) 376:1824–34. 28317458 10.1056/NEJMoa1700445

[11] Gotberg M Christiansen EH Gudmundsdottir IJ Sandhall L Danielewicz M Jakobsen L Instantaneous wave-free ratio vs. fractional flow reserve to guide PCI. *N Engl J Med.* (2017) 376:1813–23. 28317438 10.1056/NEJMoa1616540

[12] Thygesen K Alpert JS Jaffe AS Simoons ML Chaitman BR White HD Third universal definition of myocardial infarction. *Eur Heart J.* (2012) 33:2551–67. 22922414 10.1093/eurheartj/ehs184

[13] De Bruyne B Pijls NH Kalesan B Barbato E Tonino PA Piroth Z Fractional flow reserve-guided PCI vs. medical therapy in stable coronary disease. *N Engl J Med.* (2012) 367:991–1001. 22924638 10.1056/NEJMoa1205361

[14] Smits PC Abdel-Wahab M Neumann FJ Boxma-de Klerk BM Lunde K Schotborgh CE Fractional flow reserve-guided multivessel angioplasty in myocardial infarction. *N Engl J Med.* (2017) 376:1234–44. 28317428 10.1056/NEJMoa1701067

[15] Petraco R Park JJ Sen S Nijjer SS Malik IS Echavarria-Pinto M Hybrid iFR-FFR decision-making strategy: implications for enhancing universal adoption of physiology-guided coronary revascularisation. *EuroIntervention.* (2013) 8:1157–65. 10.4244/EIJV8I10A179 23256988

[16] Shuttleworth K Smith K Watt J Smith JAL Leslie SJ. Hybrid instantaneous wave-free ratio-fractional flow reserve vs. fractional flow reserve in the real world. *Front Cardiovasc Med.* (2017) 4:35. 10.3389/fcvm.2017.00035 28612008 PMC5447668

[17] Casanova-Sandoval J Fernandez-Rodriguez D Otaegui I Gil Jimenez T Rodriguez-Esteban M Rivera K Usefulness of the hybrid RFR-FFR approach: results of a prospective and multicenter analysis of diagnostic agreement between RFR and FFR-the RECOPA (REsting full-cycle ratio comparation vs. fractional flow reserve (a prospective validation)) study. *J Interv Cardiol.* (2021) 2021:5522707. 10.1155/2021/5522707 34007248 PMC8026323

[18] Wienemann H Meyer A Mauri V Baar T Adam M Baldus S Comparison of resting full-cycle ratio and fractional flow reserve in a german real-world cohort. *Front Cardiovasc Med.* (2021) 8:744181. 10.3389/fcvm.2021.744181 35004875 PMC8740550

[19] Niizuma S Takiuchi S Okada S Horio T Kamide K Nakata H Decreased coronary flow reserve in haemodialysis patients. *Nephrol Dial Transplant.* (2008) 23:2324–8. 18234846 10.1093/ndt/gfm954

[20] Tebaldi M Biscaglia S Fineschi M Manari A menozzi m secco gg fractional flow reserve evaluation and chronic kidney disease: analysis from a multicenter italian registry (the FREAK study). *Catheter Cardiovasc Interv.* (2016) 88:555–62. 10.1002/ccd.26364 26717890

[21] Lee JM Shin ES Nam CW Doh JH Hwang D Park J Discrepancy between fractional flow reserve and instantaneous wave-free ratio: clinical and angiographic characteristics. *Int J Cardiol.* (2017) 245:63–8. 28789845 10.1016/j.ijcard.2017.07.099

[22] Goto R Takashima H Ohashi H Ando H Suzuki A Sakurai S Independent predictors of discordance between the resting full-cycle ratio and fractional flow reserve. *Heart Vessels.* (2021) 36:790–8. 33398440 10.1007/s00380-020-01763-1

[23] Kobayashi Y Johnson NP Berry C De Bruyne B Gould KL Jeremias A The influence of lesion location on the diagnostic accuracy of adenosine-free coronary pressure wire measurements. *JACC Cardiovasc Interv.* (2016) 9:2390–9. 10.1016/j.jcin.2016.08.041 27838269

[24] De Bruyne B Pijls NH Bartunek J Kulecki K Bech JW De Winter H Fractional flow reserve in patients with prior myocardial infarction. *Circulation.* (2001) 104:157–62. 11447079 10.1161/01.cir.104.2.157

[25] Lee JM Rhee TM Choi KH Park J Hwang D Kim J Clinical outcome of lesions with discordant results among different invasive physiologic indices- resting distal coronary to aortic pressure ratio, resting full-cycle ratio, diastolic pressure ratio, instantaneous wave-free ratio, and fractional flow reserve. *Circ J.* (2019) 83:2210–21. 10.1253/circj.CJ-19-0230 31484836

